# Sustained Decrease in Laboratory Detection of Rotavirus after Implementation of Routine Vaccination — United States, 2000–2014

**Published:** 2015-04-10

**Authors:** Negar Aliabadi, Jacqueline E. Tate, Amber K. Haynes, Umesh D. Parashar

**Affiliations:** 1 Epidemic Intelligence Service, CDC; 2 Division of Viral Diseases, National Center for Immunization and Respiratory Diseases, CDC

Rotavirus infection is the leading cause of severe gastroenteritis among infants and young children worldwide (1,2). Before the introduction of rotavirus vaccine in the United States in 2006, rotavirus infection caused significant morbidity among U.S. children, with an estimated 55,000–70,000 hospitalizations and 410,000 clinic visits annually (3). The disease showed a characteristic winter-spring seasonality and geographic pattern, with annual seasonal activity beginning in the West during December-January, extending across the country, and ending in the Northeast during April-May (4). To characterize changes in rotavirus disease trends and seasonality following introduction of rotavirus vaccines in the United States, CDC compared data from CDC’s National Respiratory and Enteric Virus Surveillance System (NREVSS), a passive laboratory reporting system, for prevaccine (2000–2006) and postvaccine (2007–2014) years. National declines in rotavirus detection were noted, ranging from 57.8%–89.9% in each of the 7 postvaccine years compared with all 7 prevaccine years combined. A biennial pattern of rotavirus activity emerged in the postvaccine era, with years of low activity and highly erratic seasonality alternating with years of moderately increased activity and seasonality similar to that seen in the prevaccine era. These results demonstrate the substantial and sustained effect of rotavirus vaccine in reducing the circulation and changing the epidemiology of rotavirus among U.S. children.

NREVSS is a national laboratory-based passive reporting system for respiratory and enteric viruses, including rotavirus. Participating laboratories report weekly data to CDC, including the total number of stool samples tested for rotavirus by enzyme immunoassay and the number of specimens that tested positive. Annually, 75 to 90 laboratories report rotavirus testing data to NREVSS. A reporting year is defined as the period from July (epidemiologic week 27) to June (epidemiologic week 26) of the following year, beginning in July 2000. Rotavirus season onset is defined as the first of 2 consecutive weeks where 10% or more of specimens test positive for rotavirus. Similarly, season offset is defined as the last of 2 consecutive weeks where 10% or more of samples test positive. Peak season intensity is defined as the week with the highest proportion of tests positive for rotavirus. For analysis of season duration and peak intensity, data from all participating laboratories were included. The proportion of samples that tested positive for rotavirus and the mean decrease from the prevaccine years are reported for these data. Analyses of trends in disease were restricted to the 23 laboratories that consistently reported at least 26 weeks of data for each reporting year from July 2000 through June 2014. For this analysis, data are aggregated by week and reported as a 3-week moving average of total number of tests and rotavirus positive tests performed for the prevaccine period (2000–2006) and for each prevaccine season. Data are presented for the United States overall and for each U.S. census region.

Data from all participating NREVSS laboratories showed that with prevaccine seasons (2000–2006), median season onset was in epidemiologic week 50 (in December), peak activity was in week 9 (February/March, 43.1% positive samples) and season duration was 26 weeks. In comparison, these data showed that each of the 7 postvaccine seasons from 2007–2014 started later (if at all), had lower peak positivity for rotavirus (10.9%–27.3%), and were shorter in duration (0–18 weeks) (Table 1 and Figure 1). In the rotavirus reporting years spanning 2009–2010, 2011–2012 and 2013–2014, no seasonal onset occurred nationally, and the proportion of tests positive for rotavirus during the peak week was lower than the immediately preceding and following seasons. Examination of data for each region individually showed slight differences in seasonal onset, duration, and offset. Notably, in the South, season onset and duration varied, with some postvaccine years’ season onset and duration comparable with median values from prevaccine years. This region also had only one reporting year where no season onset threshold was reached, whereas all other regions had at least two such reporting years. Regardless of these variations, most seasons within each region showed decreased length and activity compared with prevaccine years.

Data from 23 consistently reporting laboratories demonstrated a marked decline in rotavirus testing and positivity in the postvaccine years (Table 2 and Figure 2). Overall, after vaccine introduction, the number of total tests performed as well as the number of positive rotavirus tests declined each reporting year compared with those of the prevaccine years. Furthermore, the proportion of tests that were positive for rotavirus declined from 57.8%–89.9% in each of the seven postvaccine reporting years compared with prevaccine years combined, with alternating years of lower and greater positivity rates. Similar patterns were observed when the data were examined for each region.

## Discussion

A marked and sustained decline in rotavirus activity was seen nationally in all seven rotavirus reporting years from 2007 to 2014 following the implementation of routine rotavirus vaccination of U.S. children. The decline was accompanied by changes in the predictable prevaccine seasonal pattern of rotavirus activity. The later onset and shorter duration of rotavirus seasons in the postvaccine era, including some years without a defined rotavirus season, could be a result of fewer unvaccinated, susceptible infants, resulting in reduced intensity and duration of rotavirus transmission (5). This reduced transmission of rotavirus likely also explains the declines in rates of rotavirus disease that have been seen in unvaccinated older children and even in some adult age groups in postvaccine years compared with the prevaccine era, resulting from the phenomenon known as herd immunity (6).

Biennial peaks in rotavirus activity also emerged in the postvaccine era in contrast to the annual peaks before vaccine implementation, although even the postvaccine reporting years with heavier rotavirus burden still demonstrated rotavirus activity levels that were substantially lower than those of the prevaccine years. This biennial pattern might be explained by an accumulation of a sufficient number of unvaccinated susceptible children over two successive reporting years to result in stronger rotavirus seasons every other year. Though rotavirus vaccine coverage among children aged 19–35 months has increased nationally since the vaccine was introduced, from 43.9% in 2009 to 72.6% 2013 (7), some children remain unvaccinated. In a low rotavirus reporting year, these unvaccinated children might not be exposed to wild-type rotavirus and thus remain susceptible in their second year of life. These susceptible children aged 12–23 months, together with unvaccinated infants from the next birth cohort, might form a critical mass of susceptible children sufficient to sustain more intense rotavirus transmission in alternate years.

The findings in this report are subject to at least four limitations. First, NREVSS only receives aggregate reports of the number of stool samples tested for rotavirus and the number of these that test positive, without any information on demographics or clinical features of individual patients, precluding detailed examination of these characteristics. Second, participating laboratory locations do not uniformly cover all areas of the United States, and as such regional biases might exist. Third, because testing for rotavirus does not alter clinical management of patients, testing practices might differ and affect comparability of data from site to site and year to year. Finally, any changes in rotavirus testing practices coinciding with implementation of the rotavirus vaccination program could affect interpretation of the disease trends, although the consistency of the declines in rotavirus activity across all regions and years argues against changes in testing being the main cause of the decline.

The declines in rotavirus activity seen in NREVSS data after vaccine introduction are supported by other U.S. studies showing declines in laboratory-confirmed rotavirus hospitalization (4) as well as reductions in outpatient visits, emergency room visits, acute gastroenteritis, and rotavirus-coded hospitalizations (8). During 2007–2011 more than 176,000 hospitalizations, 242,000 emergency department visits, and 1.1 million outpatients visits due to diarrhea were averted, resulting in costs savings of $924 million over this 4-year period (9). Given the sustained decline in rotavirus activity observed in the NREVSS data through 2014, we would expect additional medical visits due to diarrhea will have been prevented and additional cost savings accrued in the United States. The findings in this report are consistent with the high field effectiveness of vaccination observed in post-licensure epidemiologic studies (10). Taken together, these findings reaffirm the large public health impact of routine rotavirus vaccination in reducing the circulation of rotavirus among U.S. children.

What is already known on this topic?

Following the introduction of rotavirus vaccine in the United States in 2006, large declines have been observed in diarrhea and rotavirus hospitalizations among children aged <5 years, and onset of the rotavirus season has occurred later.

What is added by this report?

Analysis of data from the National Respiratory and Enteric Virus Surveillance System showed a marked and sustained decline in rotavirus activity nationally and regionally for the seven rotavirus reporting years from 2007 to 2014 following the implementation of routine rotavirus vaccination of U.S. children. In addition to rotavirus seasons with later onset and shorter duration, a biennial pattern of rotavirus activity emerged in the postvaccine era, with years of low activity and highly erratic seasonality alternating with years of greater activity and seasonality similar to those in the prevaccine era.

What are the implications for public health practice?

These findings reaffirm the large public health impact of routine rotavirus vaccination in reducing the circulation of rotavirus in U.S. children.

## Figures and Tables

**FIGURE 1:**
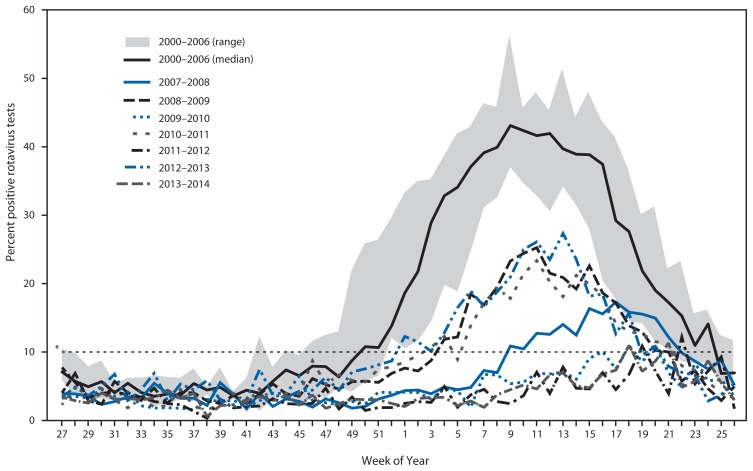
Rotavirus season duration and peak activity by reporting years (prevaccine 2000–2006 and postvaccine 2007–2011), NREVSS data — United States, 2000–2014 * Dashed line indicates the 10% threshold of numbers of positive test results, which is used to determine onset and offset of a rotavirus season.

**FIGURE 2:**
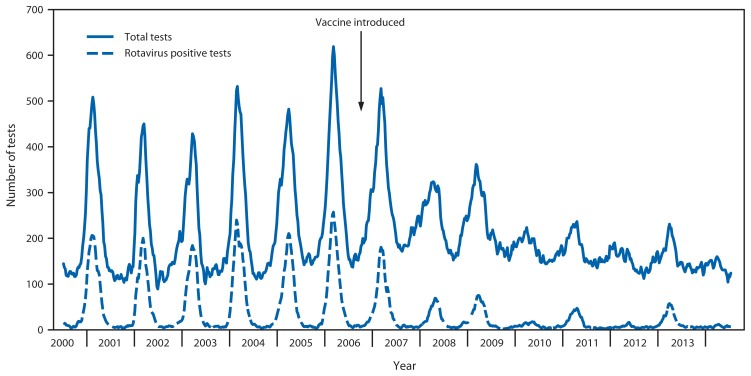
Total and positive rotavirus tests, NREVSS data — United States, 2000–2014

**TABLE 1:** Rotavirus season onset, peak activity, offset, and duration, by region — National Respiratory and Enteric Virus Surveillance System, United States 2000–2014

Overall	Onset (week no.)	Peak		Offset (week no.)	Season duration (no. weeks)

		(Week no.)	(% tests positive)		
2000–2006	50	9	43.1	24	26
2007–2008	9	17	17.3	21	12
2008–2009	4	11	25.3	21	17
2009–2010	NA*	18	10.9	NA	NA
2010–2011	3	11	23.4	21	18
2011–2012	NA	22	12.2	NA	NA
2012–2013	1	13	27.3	18	17
2013–2014	NA	21	11.3	NA	NA
**Northeast**					
2000–2006	2	11	45.2	23	21
2007–2008	18	18	13.9	19	1
2008–2009	7	11	20.1	17	10
2009–2010	NA	20	13.5	NA	NA
2010–2011	6	14	23.6	18	12
2011–2012	NA	47	10.5	NA	NA
2012–2013	10	16	28.9	21	11
2013–2014	NA	23	11.0	NA	NA
**Midwest**					
2000–2006	1	9	49.0	21	20
2007–2008	6	18	27.5	25	19
2008–2009	3	10	34.0	19	16
2009–2010	NA	19	11.6	NA	NA
2010–2011	2	14	34.3	16	14
2011–2012	18	19	13.6	19	1
2012–2013	1	11	34.3	18	17
2013–2014	NA	21	6.8	NA	NA
**South**					
2000–2006	51	10	44.0	23	28
2007–2008	12	15	16.5	21	9
2008–2009	50	9	37.2	19	31
2009–2010	15	18	17.5	18	3
2010–2011	50	11	24.7	22	28
2011–2012	NA	13	12.7	NA	NA
2012–2013	49	13	28.9	18	31
2013–2014	17	21	22.1	21	4
**West**					
2000–2006	47	5	38.1	24	23
2007–2008	11	17	28.0	22	11
2008–2009	10	15	20.9	21	11
2009–2010	NA	18	11.5	NA	NA
2010–2011	7	12	19.5	21	14
2011–2012	22	22	24.1	23	1
2012–2013	1	13	25.9	23	22
2013–2014	NA	24	17.4	NA	NA

* NA indicates years in which seasonal onset and offset threshold were not reached.

**TABLE 2:** Rotavirus tests and percent rotavirus positive results from 23 continuously reporting NREVSS laboratories, by season and region — National Respiratory and Enteric Virus Surveillance System, United States 2000–2014

Season	No. tests performed	Positive test results		Decline in no. of positive tests (%)*

		No.	%	
**All regions (23 laboratories)**				
2000–2006†	12,184	3,109	25.5	NA§
2007–2008	12,544	1,130	9	63.7
2008–2009	12,322	1,312	10.6	57.8
2009–2010	9,684	447	4.6	85.6
2010–2011	9,168	817	8.9	73.7
2011–2012	8,335	315	3.8	89.9
2012–2013	8,162	893	10.9	71.3
2013–2014	7,080	342	4.8	89
**West (eight laboratories)**				
2000–2006†	4,862	1,104	22.7	NA
2007–2008	5,813	556	9.6	49.6
2008–2009	5,127	360	7	67.4
2009–2010	4,504	196	4.4	82.2
2010–2011	3,909	257	6.6	76.7
2011–2012	3,385	144	4.3	87
2012–2013	3,043	286	9.4	74.1
2013–2014	2,939	158	5.4	85.7
**South (eight laboratories)**				
2000–2006†	3,893	1,024	26.3	NA
2007–2008	3,272	281	8.6	72.5
2008–2009	3,365	490	14.6	52.1
2009–2010	2,499	181	7.2	82.3
2010–2011	2,415	241	10	76.5
2011–2012	2,251	84	3.7	91.8
2012–2013	2,228	267	12	73.9
2013–2014	1,835	144	7.8	85.9
**Midwest (six laboratories)**				
2000–2006†	3,173	885	27.9	NA
2007–2008	3,276	281	8.6	68.2
2008–2009	3,603	450	12.5	49.1
2009–2010	2,506	63	2.5	92.9
2010–2011	2,689	298	11.1	66.3
2011–2012	2,538	84	3.3	90.5
2012–2013	2,776	330	11.9	62.7
2013–2014	2,180	36	1.7	95.9
**Northeast (one laboratory)**				
2000–2006†	194	39	19.9	NA
2007–2008	183	12	6.6	68.8
2008–2009	227	12	5.3	68.8
2009–2010	175	7	4	81.8
2010–2011	150	21	14	45.5
2011–2012	161	3	1.9	92.2
2012–2013	115	10	8.7	74
2013–2014	126	4	3.2	89.6

* This represents the decline in number of positive tests as compared to the prevaccine years (2000–2006) median; that is: (median number of positive tests 2000–2006)-(subsequent year number of positive tests)/(median number of positive tests 2000–2006)

† Median data are reported for the prevaccine seasons spanning 2000–2006.

§ NA indicates the reference period, so no values are reported.
